# Imported *Hyalomma* ticks in Germany in 2018

**DOI:** 10.1186/s13071-019-3380-4

**Published:** 2019-03-26

**Authors:** Lidia Chitimia-Dobler, Sabine Schaper, Ramona Rieß, Karin Bitterwolf, Dimitrios Frangoulidis, Malena Bestehorn, Andrea Springer, Rainer Oehme, Marco Drehmann, Alexander Lindau, Ute Mackenstedt, Christina Strube, Gerhard Dobler

**Affiliations:** 1 Bundeswehr Institute of Microbiology, Neuherbergstrasse 11, 80937 Munich, Germany; 2 Department of Parasitology, Institute of Zoology, University of Hohenheim, Emil Wolff-Strasse 34, 70599 Stuttgart, Germany; 3 Public Health Office Main-Kinzig, Barbarossastrasse 24, 63571 Gelnhausen, Germany; 4 Institute for Parasitology, Centre for Infection Medicine, University of Veterinary Medicine Hannover, Buenteweg 17, 30559 Hanover, Germany; 5 Baden-Wuerttemberg State Health Office, Stuttgart, Germany

**Keywords:** *Hyalomma marginatum*, *Hyalomma rufipes*, Sheep, Horse, Human, *Rickettsia aeschlimannii*, Germany

## Abstract

**Background:**

*Hyalomma marginatum* and *Hyalomma rufipes* are two-host tick species, which are mainly distributed in southern Europe, Africa and middle-eastern Asia. They are well-known vectors of Crimean Congo hemorrhagic fever (CCHF) virus and other viruses as well as *Rickettsia aeschlimannii*. In recent years, these tick species have been found sporadically in Germany, but they do not belong to the autochthonous tick fauna in Germany.

**Methods:**

Ticks with unusual morphology were collected and sent from private persons or public health offices to involve institutions for morphological identification and further testing. All ticks identified as *Hyalomma* spp. were tested using molecular detection methods for CCHF virus, *Rickettsia* spp., *Coxiella burnetii* and *Coxiella*-like organisms, *Babesia* spp. and *Theileria* spp.

**Results:**

Thirty-five ticks with an unusual appearance or behaviour were reported to us during summer-autumn 2018. For 17 of them, the description or photos implied that they belong to the hard tick genus *Hyalomma*. The remaining 18 ticks were sent to us and were identified as adult *Hyalomma marginatum* (10 specimens) or adult *Hyalomma rufipes* (8 specimens). All ticks tested negative for CCHF virus, *Coxiella burnetii*, *Coxiella*-like organisms, *Babesia* spp. and *Theileria* spp. The screening for rickettsiae gave positive results in 9 specimens . The *Rickettsia* species in all cases was identified as *R. aeschlimannii*.

**Conclusions:**

These results show that exotic tick species imported into Germany were able to develop from the nymphal to the adult stage under appropriate weather conditions. Fifty percent of the ticks carried *R. aeschlimannii*, a human pathogen, while CCHF virus or other pathogens were not detected. Imported *Hyalomma* ticks may be the source of exotic diseases acquired in Germany.

## Background

The genus *Hyalomma* is a small genus, with 27 species that are mainly present in the Afrotropical Region and parts of the Palaearctic Region [1]. A considerable amount of work on the genus *Hyalomma*, with an important input on classification, morphology, hosts and distribution has been done by Apanaskevich and colleagues [2–6].

*Hyalomma* (*Euhyalomma*) *marginatum* Koch, 1844 is the type-species of the *H. marginatum* complex, formed by *Hyalomma isaaci*, *Hyalomma marginatum* (*sensu stricto*), *Hyalomma rufipes*, *Hyalomma turanicum* and *Hyalomma glabrum* [7]. *Hyalomma marginatum* is known as the “Mediterranean” *Hyalomma* [8] (the synonym *Hyalomma plumbeum* has been used in some Russian and eastern European literature [2, 9, 10]). *Hyalomma marginatum* has a large geographical distribution, ranging from southern Europe and North Africa to the Ukraine and southern Russia and the Middle East [2]. Like some other *Hyalomma* species, especially of the *H. marginatum* complex, *H. marginatum* is known to be a vector of a wide variety of pathogens of medical and veterinary importance, including Crimean Congo hemorrhagic fever (CCHF) virus [8, 10], West Nile, Thogoto, Dhori and other viruses [10], as well as *Rickettsia aeschlimannii* [11, 12], *Babesia caballi* and *Theileria annulata* [8, 13]. Petney et al. [14] reviewed the tick species in Germany and found a few previous reports of *H. marginatum*, but in the majority of these cases the identification remained uncertain. A more recent study georeferenced ixodid ticks in Germany and reported one location where *H. marginatum* was identified [15, 16]. In 2017, one *H. marginatum* specimen was detected on a human in Tübingen, Federal State of Baden-Württemberg [17].

*Hyalomma rufipes* Koch, 1844 known as “the hairy *Hyalomma*” or “the coarse bont-legged *Hyalomma*” [8, 18], was considered a subspecies of *H. marginatum* [19, 20], but is currently accepted as a valid species [2]. *Hyalomma rufipes* is the most widespread *Hyalomma* species in Africa, but is also present in Greece, Turkey, Russia, Iraq, Syria, Pakistan, Egypt (Nile Valley), Yemen, Oman and northern China [8, 21–24]. Both larvae and nymphs of *H. marginatum* and *H. rufipes* use small mammals and birds as hosts, while adults are mainly found on cattle, sheep, goats, wild ungulates and horses [8, 23]. As some other *Hyalomma* species, *H. rufipes* is known to be a vector of CCHF virus [8, 18, 25] as well as of *Rickettsia conorii* [8, 18], *R. aeschlimannii* [26–28], *Anaplasma marginale* and *Babesia occultans* [8, 18]. Some authors implicated *Hyalomma* species in tick facial paralysis in humans [29, 30]. Larvae and nymphs of *H. rufipes* have been occasionally found on migratory birds in some European countries (e.g. the Netherlands and Norway) [31]. One *H. rufipes* specimen was described recently in Germany near Frankfurt, Federal State of Hesse [32]. Hoffman et al. [33] detected Alkhurma hemorrhagic fever virus RNA in immature *H. rufipes* ticks infesting northward migratory birds caught in the North Mediterranean Basin.

However, probably due to the current climatic conditions, no permanent *Hyalomma* populations have been recognized in northern or central Europe so far. Here, we report 18 imported specimens of *H. marginatum* and *H. rufipes* in Germany in 2018. The individual ticks were tested for various pathogens known to be carried by these two *Hyalomma* species.

## Methods

### Tick collection and identification

**Fig. 1:**
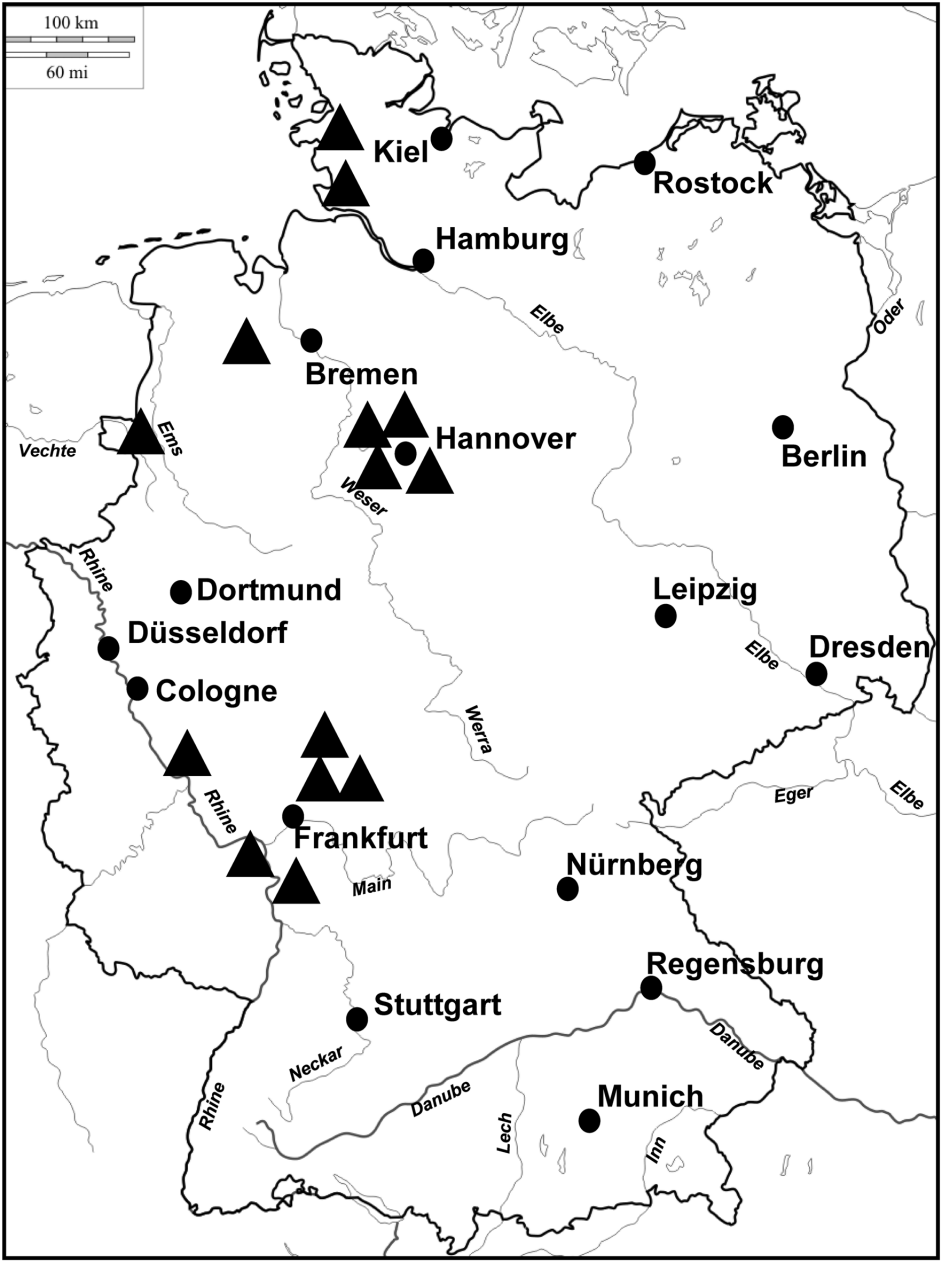
Distribution of introduced *Hyalomma* spp. in Germany, 2018

Ticks were collected from sheep, horses, a human, a house, and from one unknown site, in different locations and districts in Germany, from June to October 2018 (Table 1, Fig. 1). Ticks were shipped as individual specimens by the collecting persons directly or *via* public health offices to our laboratories. These ticks were further analysed in the present study. They were identified by morphological characters according to Apanaskevich & Horak [2]. In addition, some other collected ticks, not available for shipment, were included in this study and their identification as *Hyalomma* were based on photos sent by the animal owner.

**Table 1:** *Hyalomma* spp. collection samples and detected pathogens in Germany, 2018

Collection date	Locality and district	*Hyalomma* spp.	Stage	Host	Pathogen				
					CCHF virus	*Rickettsia aeschlimannii*	*Coxiella burnetii*	*Coxiella*-like	*Babesia*/*Theileria* spp.
26 June	Wächtersbach, Hesse	*H. marginatum*	Female	Sheep	−	+	−	−	−
30 July	Wardenburg, Lower Saxony	*H. marginatum*	Male	Horse	−	−	−	−	−
30 July	Hannover, Lower Saxony	*H. rufipes*	Female	Horse	−	−	−	−	−
05 August	Wächtersbach, Hesse	*H. marginatum*	Male	Horse	−	+	−	−	−
20 August	Hannover, Lower Saxony	*H. rufipes*	Female	Horse	−	+	−	−	−
20 August	Lützelhausen, Hesse	*H. rufipes*	Male	Horse	−	−	−	−	−
24 August	Hannover, Hesse	*H. marginatum*	Male	Car	−	+	−	−	−
13 September	Heiligenberg, Baden-Württemberg	*H. marginatum*	Male	Horse	−	−	−	−	−
22 August	Neuenhaus, Lower Saxony	*H. marginatum*	Female	Horse	−	+	−	−	−
21 August	Koblenz, Rhineland-Palatinate	*H. marginatum*	Female	Horse	−	−	−	−	−
10 September	Borgdorf-Seedorf, Schleswig-Holstein	*H. rufipes*	Male	Horse	−	−	−	−	−
23 August	Hohenaspe, Schleswig-Holstein	*H. marginatum*	Male	Horse	−	−	−	−	−
04 September	Fechenheimer Aue, Hesse	*H. marginatum*	Female	House	−	+	−	−	−
11 September	Winsen/Aller, Lower Saxony	*H. rufipes*	Female	Unknown	−	−	−	−	−
18 August	Saulheim, Rhineland-Palatinate	*H. rufipes*	Female	Horse pasture	−	+	−	−	−
13 October	Neuenkirchen, Lower Saxony	*H. rufipes*	Male	Horse	−	+	−	−	−
20 October	Wessel, North Rhine Westphalia	*H. marginatum*	Female	Horse	−	−	−	−	−
13 October	Mörsdorf, Rhineland-Palatinate	*H. rufipes*	Male	Horse	−	+	−	−	−

*Key*: +, present; −, absent

### Nucleic acid extraction and PCR

Total nucleic acid was extracted using MagNA Pure LC RNA/DNA Kit (Roche, Mannheim, Germany) in a MagNA Pure LC instrument (Roche) according to the manufacturer’s instructions. The extracted total nucleic acid was stored at -80 °C until use.

Ticks were tested for CCHF virus using a previously published real-time RT-PCR [34], *Rickettsia* spp. DNA using a pan-*Rickettsia* real-time PCR to amplify part of the *gltA* gene [35], followed by a 23S-5S intergenic spacer region PCR [36] to identify the *Rickettsia* species and an *ompA* PCR [37] and *ompB* PCR [38] for further molecular characterization. Furthermore, the ticks were tested for *Babesia* spp. and *Theileria* spp. using a conventional PCR amplifying part of the *18S* rRNA gene [39]. Additionally, by real-time PCR and conventional PCR, respectively, ticks were tested for the occurrence of *Coxiella burnetii* and *Coxiella*-like organisms as described earlier [40, 41].

### Sequence analysis of rickettsial *ompA*, *ompB* and 23S intergenic spacer region

The 23S intergenic spacer region amplicon sequences (334 bp) and the partial *ompA* sequences were compared to sequences from GenBank using the nucleotide blast algorithm. A phylogenetic tree based on the partial *ompB* sequences was generated using the maximum-likelihood (ML) method of Mega v.5.0 [42]. Best fitting substitution models were determined with the Akaike information criterion using the ML model test implemented in MEGA v.5.0. Support for the topologies was tested by bootstrapping over 1000 replicates and gaps were excluded from the comparisons. The substitution model was GTR + I. Sequences from *R. aeschlimannii* available on GenBank (HM050278.1, AF123705.1, KU961544.1, KU723521.1, MF002557.1, KT318745.1) were included to compare the newly generated sequences. Two sequences of *R. helvetica* (AF 123725.1, GU 324465.1) were used as an outgroup.

## Results

A total of 18 tick specimens were received in our laboratories and identified as *H. marginatum* (5 females and 5 males) and *H. rufipes* (4 females and 4 males) (Table 1). Ticks were found in locations in western Germany, from the northern part of the Federal State of Baden-Württemberg along the Federal States of Hesse, Rhineland-Palatine to Lower Saxony and Schleswig-Holstein (Fig. 1).

The molecular testing of the ticks for potential pathogens of both species for CCHF virus, *C. burnetii*, *Coxiella*-like organisms, *Babesia* spp. and *Theileria* spp. were negative. The pan-Rick PCR tested positive for rickettsiae in 5 out of the 10 *H. marginatum* and 4 out of 8 *H. rufipes*. The amplification of the 23S-5S intergenic spacer region, *ompA* (*ompA*1 and *ompA*4) and *ompB* fragments with specific PCRs identified *R. aeschlimannii*.

**Fig. 2:**
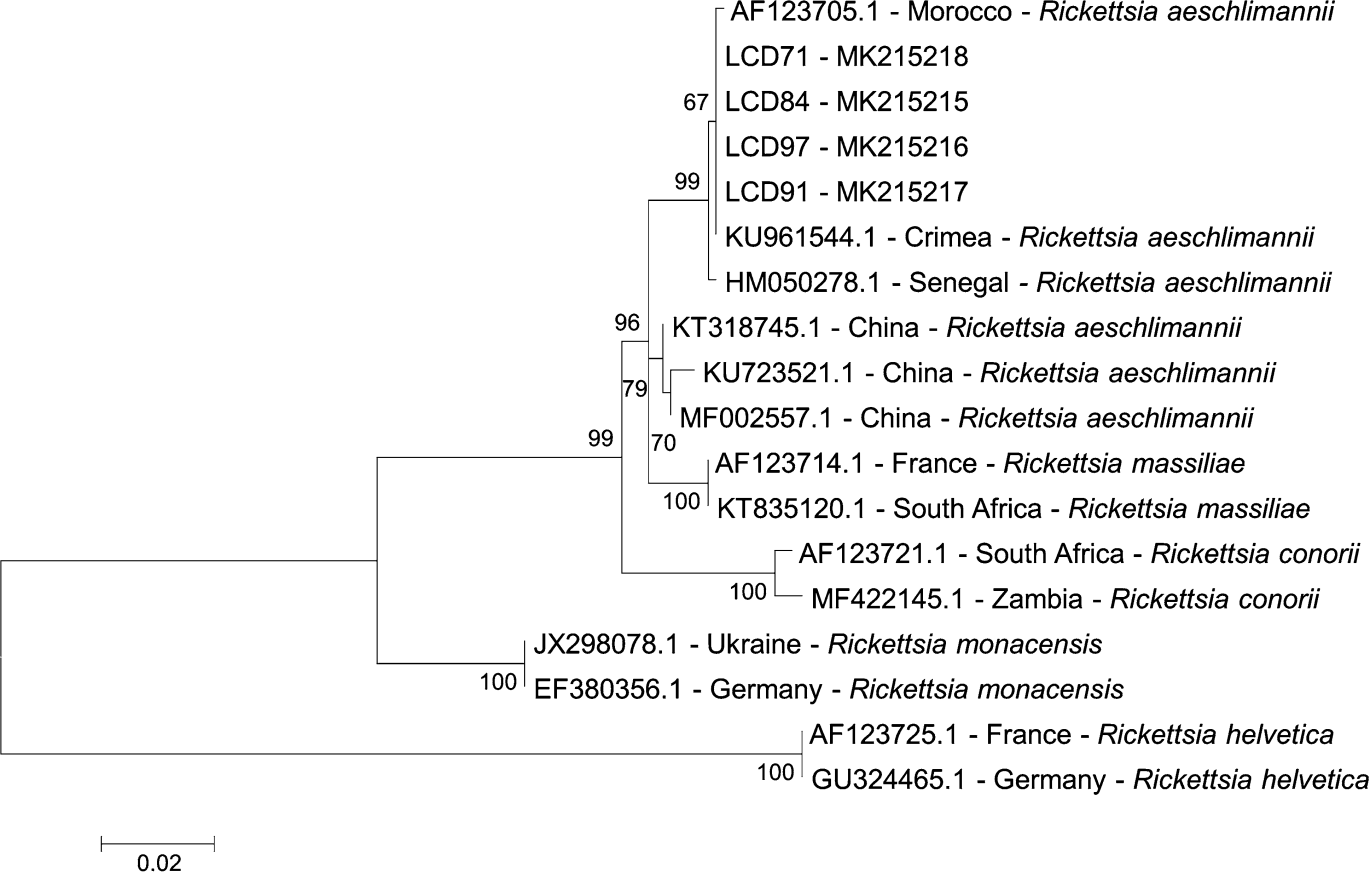
Maximum likelihood based on partial *ompB* sequences (776 nucleotides)

All nine *Rickettsia* spp. positive panRick PCR samples were further studied by amplifying and sequencing different gene fragments. 23S gene fragments were obtained and sequenced for all nine samples, *ompA* fragments for six samples and *ompB* fragments for four samples. The obtained sequences for the 23S-5S intergenic spacer region amplicon showed 100% identity with *R. aeschlimannii* sequences (GenBank: AY125016.1 and MG450333.1) on GenBank. The six *ompA*4 sequences (861 bp) were 100% identical to the *R. aeschlimannii* sequence from the strain MC16 (GenBank: U83446.1). Six out of seven *ompA*1 sequences obtained from the German samples showed a 100% identity to strains from different areas in the world (Russia, Israel, Spain, Portugal and Turkey), while one *R. aeschlimannii* sequence from a *H. marginatum* tick had a single nucleotide polymorphism at position 264 in the alignment (273 bp), which is identical to a sequence from Senegal (GenBank: HM050290.1). The four sequences obtained for the *ompB* gene (MK215215-MK215218) were 100% identical and cluster with strains from Morocco and Senegal (GenBank: HM050278.1, AF123705.1) (Fig. 2).

## Discussion

Here we report an unusually high introduction of *Hyalomma* spp. into Germany. From the 35 recorded *Hyalomma* ticks, 18 specimens were received and identified as *H. marginatum* (10 specimens) and *H. rufipes* (8 specimens). The others (17 ticks) were identified based on photos. Detection of *Hyalomma* ticks in central Europe and also northern Europe, i.e. outside of the known areas of distribution of these tick species, is not totally new. *Hyalomma marginatum* was described for the first time in northern Europe in 1939 on the Island of Bornholm [43]. Later they were described on several occasions in Finland, Sweden and Norway [44–46]. In Poland, four specimens of unfed *H. marginatum* males were found in Bytom, Upper Silesia, in June 1935 (1 specimen) and June 1943 (3 specimens), which are archived in Bytom’s museum collection, Upper Silesia [47]. In Germany, four reports of *Hyalomma* ticks are known to the best of our knowledge. Two cases of adults, one *H. rufipes* male collected from a horse [32] and one *H. marginatum* female collected from a human [17] in the Frankfurt area and Tübingen, respectively, were reported in Germany, and two other reports date from 2007 and 2011 [15, 48]. Therefore, in 2018, the reporting of 35 putative and identified ticks of the genus *Hyalomma* and the final confirmation of identification and analysis of 18 specimens in Germany are exceptional.

All reported and confirmed tick findings were located in western Germany. Ticks were found along the Rhine River and continuing up to Schleswig-Holstein in northern Germany. This implies that the main route of introduction was most likely *via* the western migratory route of birds from West Africa *via* Spain and France to Scandinavia.

While in Scandinavia nymphal ticks were collected from migrating birds, all *Hyalomma* specimens described and tested in 2018 in Germany were adult ticks sampled from large animals or humans. The immature stages of *H. marginatum* are commonly found on migratory passerine birds [10], which may transport these ticks over long distances [49–52]. Up to 21% of birds migrating from Africa to the United Kingdom were infested with *H. marginatum* nymphs [53]. Therefore, it can be estimated that every year hundreds of thousands of immature *Hyalomma* ticks are transported *via* migratory birds into or over central Europe during the spring migration of birds from southern Europe and Africa. *Hyalomma marginatum* also attacks humans [54]. In a report, *Hyalomma* species were transported from one continent to the other by humans [55].

Usually, only few of these imported ticks seem to develop into the mature stage and, so far, no established populations of *Hyalomma* ticks in central Europe are known. However, the weather conditions in 2018 in Germany allowed the molting into adult ticks, and these adult stages were subsequently found on animals, humans or as questing ticks as described above. According to the German National Weather Service, 2018 was the warmest year ever recorded since the beginning of weather recording in 1881. In addition, 2018 was the second driest year since 1881 [56]. Only the year 1911 was drier than 2018 [56]. The combination of dry and hot conditions probably favored the development and molting of imported nymphs of *Hyalomma* ticks into adults.

An accurate modelling has hypothesized that the current northern distribution limit for this tick species should be 47°N [57]. Interestingly, the same authors have hypothesized the expansion of the geographical areas, where *H. marginatum* could complete the life-cycle up to some areas in Germany and the Netherlands by the 2050s, if not before [58, 59]. Despite these forecasts, adult *Hyalomma* ticks attached to mammalian hosts in areas further north of the forecasted hypothetical geographical limit were recently reported [17, 32]. These findings confirm and even anticipate the forecasts of the models mentioned above [58, 59].

Ticks belonging to the *H. marginatum* complex are known to transmit viral and bacterial agents with the potential to cause diseases of variable severity in humans. Among the viruses, CCHF virus is of greatest medical importance. *Hyalomma marginatum* is the most important vector of this virus in the Mediterranean area [8, 10]. Besides CCHF virus, a number of other viruses have been detected in *Hyalomma* ticks, among them Wad Medani virus, Bahig virus, Matruh virus and Wanowrie virus [60]. The pathogenicity of these arboviruses is unknown. In the Ukraine, the European subtype and the Siberian subtype of tick-borne encephalitis (TBE) virus were isolated in several instances from *H. marginatum* [61]. However, the biological role of *H. marginatum* to support the natural transmission cycle under the ecological conditions of the Ukraine and the medical importance of this tick species for the transmission of TBE virus to humans and animals (with the potential alimentary infection by milk and cheese) are unknown. In several instances West Nile virus was isolated from *H. marginatum* [62, 63]. However, similar to TBE virus, the role of ticks in the natural transmission cycle and in the transmission to humans and animals needs to be further elucidated. In presumably *H. rufipes* nymphs collected from migratory birds on the Island of Capri, Italy, and in Andikithira, Greece, Alkhumra virus, a flavivirus of the tick-borne flavivirus group, was detected [33]. This virus causes a severe form of hemorrhagic fever which occurs mainly at the Arabian Peninsula but was also detected in travellers returning from Egypt [64].

Another pathogen associated with ticks of the genus *Hyalomma* is *R. aeschlimannii* [11, 12, 65, 66], a member of the spotted fever group (SFG). *Rickettsia aeschlimannii* was first described in *H. marginatum* ticks in Morocco [67]. Later it was detected in the same tick species in Europe [66, 68] and in several African countries, such as Niger, Mali and Senegal [26]. *Rickettsia aeschlimannii* was also identified by molecular means in ticks of the *H. marginatum* complex collected from birds in Pakendorf and Zerbst, Saxony-Anhalt, Germany, in May 2007 [48]. However, no identification of the tick to species level was done. In a recent study on SFG rickettsiae in ticks from migratory birds, almost 50% of ticks of the genus *Hyalomma* found as immature stages on birds in Italy and Greece were infected with rickettsiae. Among 657 collected ticks of the genus *Hyalomma*, 230 ticks (35%), exclusively larvae and nymphs, were found positive for *R. aeschlimannii*. Our data are comparable with these data. However, our ticks were exclusively adult stages. Here, 5/10 (50%) *H. marginatum* were found positive and 4/8 (50%) *H. rufipes* (Table 1) contained *R. aeschlimannii* DNA. *Rickettsia aeschlimannii* was detected in non-engorged adult ticks. These results confirm transstadial transmission of *R. aeschlimannii* from the nymphal to adult stage and show the potential risk of transmission of this rickettsial species to humans and animals by the imported ticks. It is also unclear whether large animals may play a role in the transmission cycle of this rickettsial species and whether other tick species, mainly of the *Ixodes ricinus* complex, may become infected and establish a transmission cycle under central European ecological conditions. Raoult et al. [69] detected *R. aeschlimannii* for the first time in a patient, who developed symptoms after returning from Morocco.

Nine of the introduced specimens were positive for *R. aeschlimannii* showing a 100% identity with *R. aeschlimannii* sequences from GenBank for the 23S intergenic spacer region (GenBank: AY125016.1 and MG450333.1), two *ompA* fragments (GenBank: U83446.1, HM050290.1, DQ459390.1) as well as an *ompB* fragment (GenBank: AF123705.1, HM050278.1). Due to the high homology of the analyzed sequences of the rickettsial gene fragments, a phylogenetic analysis of the *R. aeschlimannii* sequences and the ticks is difficult. However, the occurrence mainly in the western part of Germany and the closest phylogenetic relationship of *ompB R. aeschlimannii* sequences (Fig. 2) let us speculate that the main direction of introduction was along the southwestern route of bird migration.

For *C. burnetii*, the agent of Q fever, the main method of transmission is inhalation or ingestion, rather than an infective tick bite [70], although this pathogen occurs in different tick species including *Hyalomma*. In addition, tick endosymbionts (as *Coxiella*-like organisms) have been identified regularly in blood-feeding ticks [71]. However, in our study all tested specimens were negative for *C. burnetii* and *Coxiella*-like agents.

All ticks tested were found negative for *Babesia* spp. and *Theileria* spp. So far, there is only little information available on the importance of *H. marginatum* and *H. rufipes* as vectors for these two pathogen groups. *Theileria equi* was found in 9.2% and *Babesia* (*B.*) *caballi* in 1.6% of *Hyalomma* ticks in Tunisia [72]. In another study from Tunisia only 3/120 ticks tested were found positive for *B. occultans* and *Babesia* sp. Kayseri I [73]. In Somalia, none of the three *Hyalomma* species tested were found positive for *Theileria* spp. [74]. In Turkey, only one of 30 *H. marginatum* ticks was found positive for *B. occultans* [75]. These limited data show that *Hyalomma* ticks seem not to exhibit a high prevalence of piroplasms, which is in concordance with our results. However, no data on the occurrence and prevalence of *Babesia* and *Theileria* species in ticks are available for the assumed areas of origin in southwestern Europe and western Africa.

## Conclusions

As *Hyalomma* larvae and nymphs are regularly found on migratory birds, there is good reason to assume that these ticks are regularly imported as feeding nymphs by migratory birds coming from endemic areas in southern Europe and Africa to central Europe. This is an example of a tropical or sub-tropical tick species molting from the nymphal stage to the adult under favorable weather conditions outside the usual distribution area. The detection of *R. aeschlimannii* in the imported *H. marginatum* and *H. rufipes* to Germany is of importance, as it is a human pathogen.

## Abbreviations

CCHF: Crimean Congo hemorrhagic fever
PCR: reverse transcription-polymerase chain reaction
ML: maximum likelihood
TBE: tick-borne encephalitis
